# Invasive Group A Streptococcal Disease in Persons Experiencing Postpandemic Homelessness in Canada

**DOI:** 10.1001/jamanetworkopen.2025.57932

**Published:** 2026-02-10

**Authors:** Caroline Kassee, Halima Dabaja-Younis, Lucie Richard, Alyssa R. Golden, Zoe Zhong, Vanessa Allen, Huda Almohri, Irene Armstrong, Mahin Baqi, Kevin R. Barker, Sergio Borgia, Aaron Campigotto, Sumon Chakrabarti, Wayne L. Gold, Rachel K. Hink, Christopher Kandel, Ian Kitai, Julianne Kus, Liane Macdonald, Matthew P. Muller, Jeya Nadarajah, Krystyna Ostrowska, Daniel Ricciuto, David Richardson, Medina Saffie, Michael Silverman, Manal Tadros, Monali Varia, Stephen W. Hwang, Irene Martin, Allison McGeer

**Affiliations:** 1 Department of Microbiology, Sinai Health, Toronto, Ontario, Canada; 2 Infection Prevention and Control Unit, Rambam Health Care Campus, Haifa, Israel; 3 MAP Centre for Urban Health Solutions, Unity Health Toronto, Toronto, Ontario, Canada; 4 National Microbiology Laboratory, Public Health Agency of Canada, Winnipeg, Manitoba, Canada; 5 Lifelabs, Toronto, Ontario, Canada; 6 Toronto Public Health, Toronto, Ontario, Canada; 7 William Osler Health System, Brampton, Ontario, Canada; 8 Trillium Health Partners, Mississauga, Ontario, Canada; 9 The Hospital for Sick Children, Toronto, Ontario, Canada; 10 University Health Network, Toronto, Ontario, Canada; 11 Michael Garron Hospital, Toronto East Health Network, Toronto, Ontario, Canada; 12 Public Health Ontario, Toronto, Ontario, Canada; 13 Unity Health, Toronto, Ontario, Canada; 14 Oak Valley Health, Markham, Ontario, Canada; 15 Lakeridge Health, Oshawa, Ontario, Canada; 16 Joseph Brant Hospital, Burlington, Ontario, Canada; 17 Schulich School of Medicine and Dentistry, Western University, London, Ontario, Canada; 18 Region of Peel–Public Health, Brampton, Ontario, Canada

## Abstract

**Importance:**

Both invasive group A streptococcal (iGAS) infections and the number of persons experiencing homelessness (PEH) are increasing. Protection of PEH from the burden of iGAS infections requires understanding of its epidemiology.

**Objective:**

To assess whether the resurgence of iGAS infections after the COVID-19 pandemic included PEH.

**Design, Setting, and Participants:**

This cross-sectional study of population-based iGAS surveillance used Canada’s National Microbiology Laboratory for *emm* typing and Statistics Canada and point-in-time counts to identify denominators. Participants included 503 persons with iGAS infections from January 1, 2022, to December 31, 2023, in the Toronto and Peel Region, Canada (population, 4.5 million).

**Main Outcomes and Measures:**

The main outcome was disease incidence among PEH over time and compared with housed persons. Secondary outcomes were differences in risk factors, presentation, disease severity, and infecting *emm* types between PEH and housed persons.

**Results:**

Ninety iGAS cases occurred among PEH (median age, 47.0 years [IQR, 37.7-59.5 years]; 66 men [73.3%]) and 413 occurred among housed adults (median age, 58.9 years [IQR, 42.1-73.3 years]; 259 men [62.7%]). iGAS incidence among PEH increased from 270.4 (95% CI, 184.5-383.2) per 100 000 per year in 2022 to 451.2 (95% CI, 348.2-575.7) per 100 000 per year in 2023 (incidence rate ratio [IRR], 1.67; 95% CI, 1.06-2.69), not significantly different than the increase from 3.4 to 7.0 per 100 000 per year among housed persons (IRR, 2.05; 95% CI, 1.67-2.52). iGAS incidence overall was 70.7-fold higher (95% CI, 56.3-fold to 88.7-fold) among PEH than housed persons. Compared with housed adults, PEH were less likely to be immunocompromised (adjusted odds ratio [AOR], 0.29; 95% CI, 0.11-0.73) and were more likely to be persons who inject drugs (AOR, 5.06; 95% CI, 2.79-9.19), to have nonintact skin (AOR, 4.16; 95% CI, 2.45-7.04), and to have iGAS presenting as soft tissue infection (AOR, 1.64; 95% CI, 1.02-2.64). PEH were less likely to die of iGAS than housed adults (AOR, 0.33; 95% CI, 0.12-0.95). Overall, *emm*1 and *emm*12 caused 33.7% of iGAS cases (137 of 406) among housed persons, but only 2.2% (2 of 90) among PEH; in contrast, isolates with *emm* types 49, 74, 80, 82, and 92 caused 77.8% of iGAS cases (70 of 90) among PEH, but only 34.2% (139 of 406) among housed persons (*P* < .001). The *emm* types frequently causing iGAS infections among PEH also caused iGAS infections among housed persons and were too highly clonal to assess transmission risk.

**Conclusions and Relevance:**

In this cross-sectional study, the post–COVID-19 pandemic resurgence of iGAS infections occurred among both PEH and housed adults, although the incidence among PEH was 70.7-fold greater. Risk factors, clinical presentations, outcomes, and infecting strains were very different. Improved iGAS protection for PEH, such as vaccines, is needed.

## Key Points

**Question:**

During the post–COVID-19 pandemic resurgence, how did invasive group A streptococcal (iGAS) infection compare between persons experiencing homeless (PEH) and housed persons?

**Findings:**

This cross-sectional study demonstrated that iGAS increased among both PEH and housed persons; PEH were less likely to be immunocompromised, but more likely to be persons who inject drugs, to have nonintact skin, and to present with soft tissue infection. iGAS was 70.7-fold more common among PEH than housed persons and *emm* types causing disease were different between the groups.

**Meaning:**

The post–COVID-19 pandemic iGAS resurgence included PEH despite very different iGAS epidemiology between PEH and housed persons.

## Teaser

This cross-sectional study compares the resurgence of invasive group A streptococcal (iGAS) infections after the COVID-19 pandemic between persons experiencing homelessness and housed persons.

## Introduction

Group A *Streptococcus* (GAS; *S pyogenes*) is a gram-positive bacterium and obligate human pathogen that commonly colonizes the throat and skin. GAS most often causes mild infections (eg, pharyngitis, impetigo) but can also cause bacteremia, necrotizing fasciitis, and streptococcal toxic shock syndrome, all of which are associated with high morbidity and mortality.^1^ The most antigenic cell surface protein of GAS is the M protein, encoded by the *emm* gene, with variability among strains described by *emm* types, and, more recently, by *emm-*cluster types.^2^

Persons experiencing homelessness (PEH) and persons who inject drugs (PWID) are disproportionally affected by invasive GAS (iGAS) infection.^3,4,5^ These infections may have contributed to the increasing iGAS incidence reported in multiple jurisdictions in the years preceding the COVID-19 pandemic.^6,7^ In 2022, after the COVID-19 pandemic, many jurisdictions reported a marked resurgence in the incidence of iGAS infection, particularly among children.^8,9,10^

We analyzed data from population-based surveillance for iGAS infections in Toronto and Peel Region in Ontario, Canada, in 2022 and 2023 to evaluate the incidence, clinical features, and microbiologic characteristics of iGAS infections among PEH, as compared with housed persons, during this resurgence.

## Methods

This cross-sectional study was approved with a waiver of informed consent in accordance with established ethical guidelines for human research involving medical record reviews by the research ethics boards of participating institutions (Baycrest, Halton Health Sciences, Hospital for Sick Children, Humber River Hospital, Joseph Brant Hospital, Lakeridge Health, MacKenzie Health, Oak Valley Health, Sinai Health System, Southlake Health, Sunnybrook Health Sciences Centre, University Health Network, Salvation Army Toronto Grace Hospital, Scarborough Health Network, Toronto East Health Network, Trillium Health Partners, Unity Health Toronto, West Park Healthcare Centre, William Osler Health System, and Women’s College Hospital). This study is reported following the Strengthening the Reporting of Observational Studies in Epidemiology (STROBE) reporting guideline for cross-sectional studies.

### Population-Based Surveillance

The Toronto Invasive Bacterial Diseases Network (TIBDN) has conducted population-based surveillance for iGAS infections among residents of Toronto and Peel Region since 1992 (population in 2023, 4.7 million).^11^ This analysis used iGAS cases diagnosed between January 1, 2022, and December 31, 2023. We defined residence within the surveillance area by postal code and counted PEH admitted to hospitals in the surveillance area as residents. Laboratory-based surveillance included all 28 hospitals providing care to residents and all 25 laboratories processing sterile site cultures from the population area. Laboratory personnel notified the TIBDN office whenever GAS was isolated from sterile site specimens. We conducted annual audits to ensure complete reporting.

### Laboratory Procedures

Isolates were sent to the TIBDN laboratory at Mount Sinai Hospital in Toronto for confirmation of *S pyogenes*; *emm* typing was performed at Canada’s National Microbiology Laboratory using whole genome sequencing (WGS) and the WGS Analysis and Detection of Molecular Markers pipeline as previously described.^12^ For *emm* types where the proportion of all isolates causing iGAS infections among PEH was at least twice that of isolates causing iGAS infections among housed adults, we performed core single nucleotide variant (SNV) phylogenetic analysis using the SNVPhyl pipeline^12,13^ and aimed to identify genomic clusters using ClusterPicker, version 1.2.5.^14^ Antimicrobial susceptibility testing was performed following Clinical Laboratory Standard Institute guidelines and/or by prediction from WGS.^12,15^

### Data Collection and Definitions

We obtained clinical and demographic data by medical record review, with the primary site of infection determined based on attending physician documentation. Underlying comorbidities and reasons for and outcomes of health care visits in the 7 days prior to iGAS diagnosis were recorded as documented in medical records. iGAS infection was defined according to the Canadian case definition: systemic illness associated with the isolation of GAS from a normally sterile body fluid or site or from nonsterile specimens if associated with streptococcal toxic shock syndrome or necrotizing fasciitis.^16^ Immunosuppression comprised both immunocompromising conditions and long-term receipt of immunosuppressive therapy, as previously described.^17^ Streptococcal toxic shock syndrome was defined using the 2010 US Centers for Disease Control case definition.^18^ iGAS-related mortality was defined as death occurring during hospitalization and within 30 days of disease onset.

### Statistical Analysis

We conducted data analyses in R, version 4.2.0 (R Project for Statistical Computing), with continuous variables compared using Wilcoxon rank sum tests, and proportions compared using χ^2^ or Fisher exact tests. Clinical and microbiologic features of iGAS were compared between PEH and housed persons using odds ratios (ORs) and Wald 95% CIs adjusted for age and sex. Multivariable logistic regression was used to assess the association between homelessness and death, adjusting for variables associated with iGAS case fatality consistently reported in published literature, and those identified in age- and sex-adjusted analyses in our data.^19,20,21,22,23,24,25,26^ An exploratory model adjusted for variables associated with mortality identified by the US Active Bacterial Core (ABC) surveillance.^21^

Overall population estimates were obtained from Statistics Canada, with the housed population estimated as (total population − PEH population).^27^ Because we assumed that homelessness itself was the most important risk factor for iGAS infection, and because the incubation time for GAS is short, we interpolated data from point-in-time counts conducted in Toronto and Peel Region in 2021 and 2024, assuming a steady monthly percentage population increase over time between the counts to estimate the population of PEH.^28,29,30^ Point-in-time counts use a validated, standardized method for estimating the daily prevalence of PEH (see eMethods and eTable 1 in Supplement 1, which also presents results using an alternate denominator of the estimated number of PEH over a 1-year period). The 95% CIs for incidence rates and incidence rate ratios (IRRs) were estimated using exact Poisson methods (*epitools* package, R). We used an exponential mean model to assess whether there was interaction between year of observation and population group. All *P* values were 2-sided, with statistical significance set at *P* < .05.

## Results

### Incidence

**Figure 1.**
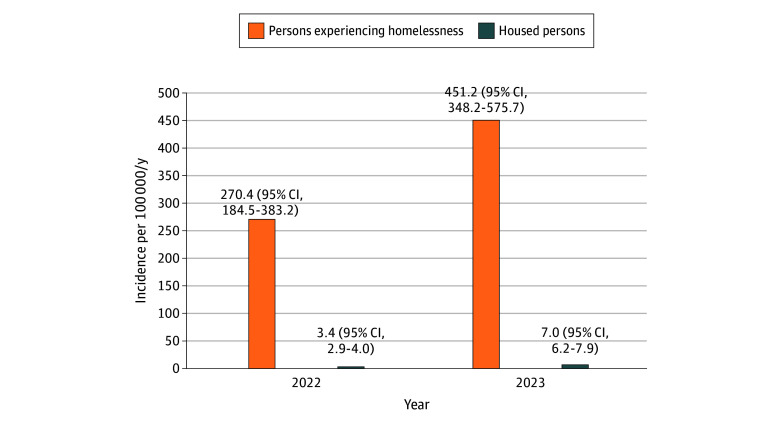
Annual Incidence of Invasive Group A Streptococcal Infection in Adults, by Housing Status, Toronto and Peel Region, January 1, 2022, to December 31, 2023

Over the 2-year surveillance period, 558 iGAS cases were identified: 55 among children (<16 years) and 503 among adults. All 90 iGAS cases among PEH occurred among adults (representing 17.9% of adult iGAS cases; median age, 47.0 years [IQR, 37.7-59.4 years]; 66 men [73.3%] and 24 women [26.7%]). A total of 413 iGAS cases occurred among housed adults (median age, 58.9 years [IQR, 42.1-73.3 years]; 259 men [62.7%] and 154 women [37.3%]). The estimated iGAS incidence in PEH increased from 270.4 (95% CI, 184.5-383.2) per 100 000 per year in 2022 to 451.2 (95% CI, 348.2-575.7) per 100 000 per year in 2023 (IRR, 1.67; 95% CI, 1.06-2.69), compared with incidence rates of 3.4 (95% CI, 2.9-4.0) per 100 000 per year in 2022 and 7.0 (95% CI, 6.2-7.9) per 100 000 per year in 2023 among housed adults (IRR, 2.06; 95% CI, 1.67-2.55) (Figure 1). iGAS incidence overall was 70.7-fold higher (95% CI, 55.4-fold to 88.7-fold) among PEH than housed persons. Using an exponential-mean model of iGAS incidence, there was no statistical interaction between and year and population group.

The incidence of iGAS infection was higher among men than women, and increased with age among both PEH and housed persons (Table 1). The IRR comparing incidence among PEH and housed persons was similar for men and women, but higher for persons aged 25 to 54 years (IRR, 101.04; 95% CI, 75.09-135.94) than for persons aged 55 or older (IRR, 59.84; 95% CI, 39.24-88.21). This difference may be associated with the fact that, among younger but not older adults, PEH were more likely than housed adults to be PWID (for those aged 24-54 years, 29 of 58 [50.0%] vs 22 of 137 [16.1%]; *P* < .001; for those aged ≥55 years, 3 of 29 [10.3%] vs 9 of 230 [3.9%]; *P* = .12).

**Table 1.** Incidence of iGAS Disease Among Adults Experiencing Homelessness Compared With Housed Adults

Characteristic	Incidence of iGAS infection per 100 000/y (95% CI)		Adults experiencing homelessness vs housed adults, IRR (95% CI)
	Adults experiencing homelessness	Housed adults	
Overall	371 (299-456)	5.3 (4.8-5.8)	70.7 (56.3-88.7)
Age group, y^a^			
16-24	35 (1-196)	1.2 (0.7-2.0)	28.8 (1.4-162.7)
25-54	389 (297-500)	3.8 (3.3-4.5)	101.0 (75.0-135.9)
≥55	560 (375-804)	9.4 (8.2-10.6)	59.8 (39.2-88.2)
Sex^b^			
Male	485 (354-583)	6.7 (6.0-7.6)	68.0 (51.1-89.4)
Female	264 (169-392)	3.8 (3.2-4.5)	68.5 (44.6-105.3)

Abbreviations: iGAS, invasive group A *Streptococcus*; IRR, incidence rate ratio.

^a^ There were no cases of iGAS among children experiencing homelessness.

^b^ Point-in-time counts measured gender; fewer than 4% of respondents reported being transgender, two-spirit, or nonbinary, and fewer than 1.5% reported being transgender. Medical record reviews recorded sex as reported in the hospital record, which may be either sex at birth or current gender.

### Clinical Characteristics and Risk Factors for iGAS Infection

In adults with iGAS infections, PEH were younger than housed persons (median age, 47.0 years [IQR, 37.7-59.4 years] vs 58.9 years [42.1-73.3 years]; *P* < .001) (Table 2).^17^ Compared with housed individuals, PEH were less likely to be immunocompromised (adjusted odds ratio [AOR], 0.29; 95% CI, 0.11-0.73) and more likely to have nonintact skin (AOR, 4.16; 95% CI, 2.45-7.04), to be current smokers (AOR, 3.33; 95% CI, 2.04-5.45), and to be PWID (AOR, 5.06; 95% CI, 2.79-9.19). PEH were less likely to die of iGAS infection than housed adults (AOR, 0.33; 95% CI, 0.12-0.95). Among PEH, the proportion of patients with iGAS with nonintact skin (eg, wounds and open insect bites, but not uninfected needle marks) was not different between PWID and other PEH (26 of 56 [46.4%] vs 12 of 32 [37.5%]; *P* = .42).

**Table 2.** Characteristics of iGAS Disease Among Adults Experiencing Homelessness Compared With Housed Adults

Characteristic	Adults experiencing homelessness (n = 90)	Housed adults (n = 413)	Adults experiencing homelessness vs housed adults, adjusted OR (95% CI)^a^	*P* value
Age, median (IQR), y	47.0 (37.7-59.5)	58.9 (42.1-73.3)	NA	<.001
Sex, No. (%)				
Male	66 (73.3)	259 (62.7)	NA	.05
Female	24 (26.7)	154 (37.3)		
Underlying medical conditions, No. (%)				
Diabetes	15 (16.7)	94 (22.8)	NA	.40
Pulmonary	9 (10.0)	63 (15.3)	NA	.76
Cardiac	8 (8.9)	94 (22.8)	NA	.14
Kidney	6 (6.7)	60 (14.5)	NA	.22
Liver	10 (11.1)	30 (7.3)	NA	.12
Autoimmune^b^	0	27 (6.5)	NA	.74
Immunocompromise^c^	5 (5.6)	86 (20.8)	0.29 (0.11-0.73)	.01
Chronic skin conditions^d^	5 (5.6)	36 (8.7)	NA	.31
Known substance use, No. (%)				
Alcohol use disorder	22 (24.4)	57 (13.8)	NA	.06
Current smoker	48 (53.3)	90 (21.8)	3.33 (2.04-5.45)	<.001
Injection drug use^e^	32/88 (36.4)	31/377 (8.2)	5.06 (2.79-9.19)	<.001
Infection source and risk factors, No. (%)				
Acute respiratory illness in previous 14 d	2 (2.2)	25 (6.1)	NA	.19
Infection related to health care	3 (3.3)	24 (5.8)	NA	.43
Recent soft tissue trauma	20 (22.2)	84 (20.3)	NA	.55
Nonintact skin at presentation^f^	38 (42.7)	74 (18.0)	4.16 (2.45-7.04)	<.001
Primary clinical diagnosis, No. (%)				
Soft tissue infection	55 (61.1)	203 (49.2)	1.64 (1.02-2.64)	.04
Bacteremia without focus	5 (5.6)	77 (18.6)	0.32 (0.12-0.81)	.02
Pneumonia and/or empyema	4 (4.4)	44 (10.7)	NA	.11
Upper respiratory tract infection	4 (4.4)	31 (7.5)	NA	.19
Arthritis or bursitis	7 (7.8)	31 (7.5)	NA	.80
Osteomyelitis	9 (10.0)	9 (2.2)	4.98 (1.84-13.51)	.002
Endocarditis^g^	5 (5.6)	1 (0.2)	24.42 (2.67-221.27)	.004
Other^h^	1 (1.1)	17 (4.1)	NA	.19
Severity of presentation, No. (%)				
Streptococcal toxic shock syndrome	4 (4.4)	68 (16.5)	0.27 (0.10-0.78)	.02
Necrotizing fasciitis	6 (6.7)	36 (8.7)	NA	.57
Treatment and outcome, No. (%)				
Hospitalized	77 (85.6)	375 (90.8)	NA	.53
Surgical procedure	24 (26.7)	101 (24.5)	NA	.85
Received intravenous immunoglobulin	3 (3.3)	42 (10.2)	NA	.06
Admitted to intensive care unit	15 (16.7)	113 (27.4)	NA	.07
Death	4 (4.4)	69 (16.7)	0.33 (0.12-0.95)	.04
Antimicrobial resistance of isolate, No./total No. (%)				
Erythromyin	15/90 (16.7)	55/410 (13.4)	NA	.78
Clindamycin	17/90 (18.9)	73/410 (17.8)	NA	.92
Tetracycline	22/90 (24.4)	84/405 (20.7)	NA	.52

Abbreviations: iGAS, invasive group A *Streptococcus*; NA, not applicable; OR, odds ratio.

^a^ Odds ratios were adjusted for age and sex.

^b^ Autoimmune conditions included rheumatoid arthritis (n = 14), systemic lupus erythematosus (n = 2), ulcerative colitis (n = 4), and 1 case each of mixed connective tissue disease, Behcet disease, polyangiitis, leukocytoclastic vasculitis, reactive perforating collagenosis, alopecia totalis, and undiagnosed arthritis.

^c^ Immunocompromise was defined as previously described,^17^ with the exception that cirrhosis and kidney failure were excluded.

^d^ Chronic skin conditions included eczema (n = 6), dermatitis (n = 8), psoriasis (n = 11), chronic wounds (n = 5), lichen planus (n = 2), prurigo nodularis (n = 2), hiradenitis suppurativa (n = 2), recurrent skin infections (n = 2), other pruritus (n = 1), pyoderma (n = 1), and severe intertrigo (n = 1).

^e^ Data on injection drug use were missing for 2 adults experiencing homelessness and 36 housed adults.

^f^ Nonintact skin at presentation was defined as any open wounds, excoriations, or lacerations, including nonhealed insect bites; uninfected needle prick marks were excluded.

^g^ The age- and sex-adjusted OR for the association between persons who inject drugs and endocarditis was 24.26 (95% CI, 2.59-227.30), suggesting that being a person who injects drugs, rather than housing status, was the relevant risk factor.

^h^ Other diagnoses included meningitis (n = 4; 1 person experiencing homelessness), gynecologic (n = 5; eg, tubo-ovarian abscess, postpartum endometritis), peritonitis (n = 3), pyelonephritis (n = 3), and intra-abdominal abscesses (n = 3).

### iGAS Presentation and Outcomes

PEH and housed persons were equally likely to have 1 or more health care visits for their infection prior to their admission (PEH, 12 of 90 [13.3%] vs housed persons, 65 of 413 [15.7%]; *P* = .57). Four of 12 PEH (25.0%) with prior visits were discharged from the emergency department (1 with fever alone and a diagnosis of viral infection, 1 with cellulitis discharged without documentation of an antibiotic prescription, and 2 with suspected methicillin-resistant *Staphylococcus aureus* [MRSA] soft tissue infections—1 prescribed doxycycline [isolate was tetracycline resistant] and 1 prescribed trimethoprim-sulfamethoxazole). Eight of 12 PEH (66.7%) declined hospitalization or self-discharged (4 with ≥2 emergency department visits).

PEH were more likely than housed adults to have soft tissue infections (AOR, 1.64; 95% CI, 1.02-2.64), osteomyelitis (AOR, 4.98; 95% CI, 1.84-13.51), and endocarditis (AOR, 24.34; 95% CI, 2.67-221.47 (Table 2).^17^ After adjustment for a history of injection drug use in addition to age and sex, the association between homelessness and endocarditis was no longer significant (AOR, 8.27; 95% CI, 0.80-85.45), while the association with osteomyelitis remained significant (AOR, 4.98; 95% CI, 1.78-13.51).

PEH were significantly less likely to die of iGAS infection than housed adults in both unadjusted analysis (4 of 90 [4.4%] vs 69 of 413 [16.7%]; *P* = .001) and after adjustment for age, sex, a presenting diagnosis of pneumonia, comorbidities, the presence of positive blood cultures, and *emm* type 1 or 3 (AOR, 0.32; 95% CI, 0.11-0.93).^19,20,21,22,23,24,25^ Analysis adjusted for variables associated with mortality in the ABC surveillance system yielded an AOR for death of 0.36 (95% CI, 0.15-1.11).^21^ No PEH with either necrotizing fasciitis or streptococcal toxic shock syndrome died (compared with housed adults: AOR, not estimable; *P* = .66 for necrotizing fasciitis and *P* = .78 for streptococcal toxic shock syndrome).

### Isolate Characteristics

Blood cultures were positive in 63 of 90 iGAS cases among PEH (70.0%) and 325 of 413 iGAS cases among housed adults (78.7%) (*P* = .08). The proportions of iGAS cases with clindamycin, erythromycin, and tetracycline resistance were similar in housed persons and PEH (Table 2).^17^

**Figure 2.**
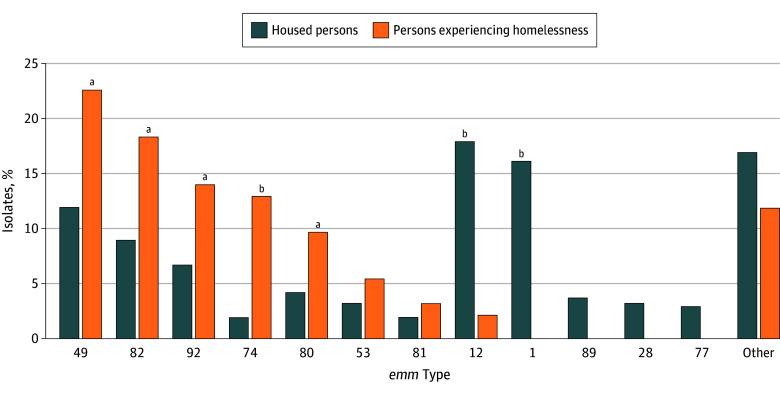
Comparison of *emm* Type Distribution Among Invasive Group A Streptococcal Infection Cases in Adults by Housing Status, Toronto and Peel Region, January 1, 2022, to December 31, 2023 Results are shown for individual *emm* types that were associated with more than 2% of total cases, with isolates of all other *emm* types grouped as “other” (complete *emm* type distribution is found in eTable 1 in Supplement 1). Housed persons had 406 of 413 cases with isolates available for *emm* typing and persons experiencing homelessness had all 90 isolates available for *emm* typing. ^a^Statistically significant difference in proportion between the 2 groups at *P* < .05 and *P* > .001. ^b^Statistically significant difference in proportion between the 2 groups at *P* < .001.

Overall, 496 of 503 isolates (98.6%) were available for *emm* typing. Compared with isolates causing iGAS in housed adults, isolates causing iGAS in PEH were much more likely to be of *emm* types 49, 74, 80, 82, and 92, and much less likely to be of *emm* types 1 and 12 (Figure 2). Isolates of *emm* types 1 and 12 caused 33.7% of iGAS cases (137 of 406) among housed persons, but only 2.2% (2 of 90) among PEH; in contrast, isolates of *emm* types 49, 74, 80, 82, and 92 caused 77.8% of iGAS cases (70 of 90) among PEH, but only 34.2% (139 of 406) among housed persons (*P* < .001). Isolates causing iGAS infections among PEH were less likely to be of *emm-*cluster A to C (ie, strains associated with pharyngitis^2^) than those causing iGAS infections among housed persons (2 of 90 [2.2%] vs 142 of 406 [35.0%]; *P* < .001). This difference remained significant when *emm*1 strains were excluded (2 of 90 [2.2%] vs 77 of 341 [22.6%]; *P* < .001). iGAS strains from PEH were more likely to be *emm*-cluster E3 (39 of 90 [43.3%] vs 94 of 406 [23.2%]; *P* < .001), D4 (17 of 90 [18.9%] vs 35 of 406 [8.6%]; *P* = .008), and Clade Y (11 of 90 [12.2%] vs 14 of 406 [3.4%] *P* = .002) than those from housed persons (cluster D4 is associated with skin infections, while E3 and Clade Y are generalist clusters).^2,31^

Phylogenetic analysis of isolates from *emm* types whose proportion among PEH was more than twice the proportion among housed persons (*emm* types 74, 80, 82, and 92) revealed very high clonality. Overall, 134 of 139 isolates occurred in 1 cluster each per *emm* type, with median SNV differences of 15 or less among isolates (Table 3). The limited number of sites that could be used to build phylogenetic trees limited their interpretation, but no visible clustering of cases was evident (eFigures 1-4 in Supplement 1). However, examination of pairs and groups of isolates with SNV differences of 2 or less identified 2 potential epidemiologically related clusters, suggesting that access to additional information might reveal transmission clusters (Table 3).

**Table 3.** Genomic and Epidemiologic Links Between Cases of iGAS Infection Caused by Strains of *emm* Types More Than Twice as Common Among PEH Than Housed Persons

*emm* type	No. iGAS cases/No. isolate groups^a^	No. of cases per group	Inter-isolate SNV differences, median (range)	Epidemiology
82	53/3	1 *emm*82.1, MLTS320 3 MLST36 49 MLST334	NA 7 (0-12) 10 (0-22)	NA All housed (no PWID); no epidemiologic links 32 housed (5 PWID); 17 PEH (7 PWID)
92	40/2	1 MLST727 39 MLST82	NA 15 (0-30)	28 Housed (4 PWID); 12 PEH (7 PWID)^b^
80	26/1	26 MLST538	5 (0-15)	17 Housed (2 PWID); 9 PEH (3 PWID)
74	20/1	20 MLST120	4 (0-9)	9 Housed (3 PWID); 11 PEH (5 PWID)^c^

Abbreviations: iGAS, invasive group A *Streptococcus*; MLST, multilocus sequence type; NA, not applicable, as a single isolate; PEH, persons experiencing homelessness; PWID, persons who inject drugs; SNV, single nucleotide variant.

^a^ Highly related isolate groups were defined as groups of isolates that were of the same MLST, and in which each isolate differed by less than 21 SNVs from at least 1 other isolate in the same grouping.

^b^ A group of 4 cases of *emm*92 with 0 to 1 SNV differences occurred over 2.5 weeks, all known to be PWID and all admitted to hospital A (4 of 4 [100%] vs 28 of 86 [32.5%] other iGAS cases among PEH admitted to hospital A; *P* = .01).

^c^ A group of 8 cases of *emm*74 with 0 to 2 SNV differences occurred over 5.5 months, 6 known to be PWID, and 7 of 8 admitted to hospital A (7 of 8 [87.5%] vs 25 of 82 [30.5%] other iGAS cases among PEH admitted to hospital A; *P* = .003).

In total, the *emm* types in the 30-valent GAS vaccine currently in development included 64.4% (58 of 90) isolates causing iGAS infections among PEH compared with 81.5% (331 of 406) of those causing iGAS infections among housed persons in our study area (*P* < .001) (eTable 2 in Supplement 1).^32^ Adding potential cross-reactive serotypes increased coverage to 86.0% (349 of 406) among housed persons and to 66.7% (60 of 90) among PEH.^33^

## Discussion

After the COVID-19 pandemic, many jurisdictions reported a marked increase in iGAS infections among children, and a lesser but significant increase in iGAS infections among adults, predominantly due to a postpandemic return of disease caused by isolates of *emm* types 1 and 12.^8,9,10,34,35,36^ In our population, the increase in iGAS incidence occurred among both the housed population and PEH, and to an indistinguishable degree. However, the increased incidence in PEH was caused by isolates of different *emm* types, with no disease due to *emm*1 and very little due to *emm*12. A recent study found very different genomic population structure and dynamics of iGAS infections in northern Australia, where GAS infections are hyperendemic, compared with southeastern Australia, where GAS infections are less common.^37^ Their modeling suggested that small populations with high transmission intensity and high population migration resulted in many lineages causing disease, with waves of replacement and without a dominant lineage, whereas larger populations with lower transmission intensity and population migration resulted in the establishment of a few dominant lineages that cycled in frequency. In our data, the distribution of *emm* types resembles the former among PEH and the latter among housed persons, suggesting that transmission dynamics among PEH and housed persons may be very different despite their geographic proximity.

As reported in other cohorts in the US, England, and Belgium, PEH with iGAS infections in our population were younger, more likely to have nonintact skin, and more likely to be PWID.^3,26,38^ The increased incidence and greater proportion of skin infections in iGAS among PEH is in part due to complications of intravenous drug use, but also associated with the increased risk of falls, cold-induced injuries, burns, assaults, infestations, and limited access to hygiene, wound care, and foot care.^39^ Data on the effect of interventions to reduce infection risk among PEH are sparse, although housing-first initiatives and access to individualized case management and better hygiene have been shown to reduce emergency department visits and hospitalizations and would be expected to reduce iGAS risk.^40,41^

A relatively small percentage of PEH (4.4%) were discharged after an initial emergency department visit prior to requiring admission on a second visit, although an additional 8.8% self-discharged from a first visit prior to returning. Two patients were discharged with therapy directed at MRSA, which was not effective against *S pyogenes*: some of the increase in iGAS infections in the last decade may be associated with patients receiving empiric therapy ineffective against *S pyogenes* because their soft tissue infections were presumed to be due to MRSA.^42^

Other studies have identified a lower case fatality rate in iGAS infections among PEH and PWID than iGAS infections among other persons.^3,26,38^ The lower case fatality rate has been attributed to infection occurring at a younger age among PEH as well as the relative absence of *emm*1 infections, which are known to be more severe. However, this association remained significant in our data despite analysis adjusting for factors known to be associated with case fatality.^19,20,21,22,23,24,25^ It is possible that our finding is a result of inadequate adjustment (eg, if common *emm* types found among PEH are less virulent than other *emm* types) or confounding. It is also possible that recurrent exposure to *S pyogenes* is associated with protection from more severe disease.

Estimates of the incidence of iGAS infection among PEH are limited. Surveillance in Brussels estimated rates ranging from 72 to 140 cases per 100 000 per year from 2010 to 2016^31^; the US ABC surveillance system estimated that the incidence in participating population areas increased from 55 per 100 000 per year in 2010 to 807 per 100 000 per year in 2022.^3,38,43^ Factors likely contributing to the variability in estimates include increases in populations of PEH and PWID over the last 15 years, as well as challenges in estimating the size of populations experiencing homelessness. Despite the variability in rates, it is clear that even the lowest estimates of risk represent an incidence markedly higher for PEH than other population groups. Planning for the implementation of GAS vaccination programs should take into account this important population group.^32,33,38^

As in other studies, and despite differences in specific *emm* types, our data demonstrate a marked absence of *emm-*cluster A to C isolates (pharyngeal specialists), and the presence of a majority of *emm-*cluster E isolates (generalists) in iGAS infections among PEHs.^26,38^ The reasons for this remain obscure. One possibility is that crowding and poor hygiene in shelters and encampments differentially increase transmission of generalist strains; the absence of exposure to children might also differentially reduce exposure to strains of some *emm* types (particularly *emm*1), which happen to be pharyngeal specialists.^44^

There is greater overlap in the *emm* types causing iGAS infections in our population and those causing iGAS infections elsewhere in Canada and the US than in those causing iGAS infections in northern Europe.^3,6,7,26,36,38,45^ The relatively low coverage of GAS strains causing iGAS infections among PEH by the 30-valent GAS vaccine in development suggest that vaccines based solely on M protein antigens may be suboptimal in this population.^4,5,45,46,47^

Core SNV phylogenetic analysis is often very useful in supporting iGAS outbreak investigations.^48,49^ However, when *emm* types are highly clonal, there may be too few core genome variant positions to reliably assess differences between isolates. As shown in our data, the *emm* types concentrated among PEH were also present among housed persons, with all isolates so closely related that we were unable to draw conclusions about the relatedness of the isolates from genomic analyses.

### Strengths and Limitations

The validated estimates of the population of PEH is a study strength; however, our estimates also have limitations. Because there are multiple reasons for increased risk of iGAS infections among PEH, it is impossible to assess the extent to which increased risk is associated with homelessness itself vs secondary to comorbidities and behavioral risk factors that persist during adjacent periods when a person is housed. Because the number of PEH over a 1-year period is 2-fold to 2.5-fold larger than the point prevalence, the incidence of disease is significantly lower if this number is used as a denominator; however, it will still be more than 25 times higher than the rate in the general population (eMethods in Supplement 1). We based our numerator data on medical record review: while the ability to identify PEH in hospital records has been validated against data collected by public health,^50^ that validation was many years ago and we may have missed PEH in this analysis. In addition, we were unable to assess the chronicity of homelessness, to assess how reliable or stable shelter use was among our population, or to assess disease incidence in “hidden homeless” populations (eg, persons temporarily staying with friends or family). Our limited power to distinguish differences means that it is not possible to be sure that the increase in iGAS incidence from 2022 to 2023 was the same as or somewhat less than that among housed persons. Nonetheless, it is clear that the postpandemic resurgence included PEH. We may not have identified PWID accurately, and there were insufficient data in medical records to permit us to describe injection drug use practices. Our data are from a single geographic area, and may not be generalizable to other populations.

## Conclusions

In this cross-sectional study of iGAS infections after the COVID-19 pandemic, the epidemiology of disease among PEH and housed persons was very different, but the magnitude of the resurgence of iGAS infections was similar. PEH were at markedly higher risk of iGAS infections than their housed counterparts, more likely to have nonintact skin and to be PWID, and more likely to present with soft tissue infection, arthritis, osteomyelitis, and endocarditis. The distribution of *emm* types of isolates causing infection was very different between the groups, with iGAS infections among PEH very rarely due to *emm-*cluster A to C strains. Despite the differences, a substantial fraction of iGAS infections among PEH was caused by types of isolates included in the 30-valent group A streptococcal vaccine in development, and vaccine programs should prioritize this population group.

## Notes

Supplement 1. **eMethods.** Estimating the Population Experiencing Homelessness in Toronto and Peel Region, 2022/2023 **eTable 1.** Estimates of the Incidence of iGAS Using a Denominator of Daily Homelessness (Point-in-Time Counts), and a Denominator of Annual Homelessness
eReferences.
**eTable 2.** Frequency of *emm* Types Among Isolates Causing iGAS in Adults, by Housing Group, TIBDN, 2022-2023, Ordered by Frequency in Persons Experiencing Homelessness, Then Housed Individuals **eFigure 1.** Maximum Likelihood Core Single Nucleotide Variant Phylogenetic Tree of *S. pyogenes emm82* Isolates of MLST334, Comprising 49 of 53 Identified *emm82* Isolates in TIBDN iGAS Surveillance Canada, January 2022–December 2023 **eFigure 2.** Maximum Likelihood Core Single Nucleotide Variant Phylogenetic Tree of *S. pyogenes emm92* Isolates of MLST82, Comprising 39 of 40 Identified *emm92* Isolates in TIBDN iGAS Surveillance Canada, January 2022–December 2023 **eFigure 3.** Maximum Likelihood Core Single Nucleotide Variant Phylogenetic Tree of *S. pyogenes emm80* Isolates of MLST538, Comprising all 26 Identified *emm80* Isolates in TIBDN iGAS Surveillance Canada, January 2022–December 2023 **eFigure 4.** Maximum Likelihood Core Single Nucleotide Variant Phylogenetic Tree of *S. pyogenes emm74* Isolates of MLST120, Comprising all 20 Identified *emm74* Isolates in TIBDN iGAS Surveillance Canada, January 2022–December 2023

Supplement 2. **Nonauthor Collaborators.** Toronto Invasive Bacterial Diseases Network Nonauthor Collaborators

Supplement 3.
Data Sharing Statement
