# Supplementary material for: Invasive Group A Streptococcal Disease in Persons Experiencing Postpandemic Homelessness in Canada
Source: JAMA Netw Open. 2026 Feb 10;9(2):e2557932. doi: 10.1001/jamanetworkopen.2025.57932 (PMC12892144; doi:10.1001/jamanetworkopen.2025.57932)
Supplement: Supplement 1. — eMethods. Estimating the Population Experiencing Homelessness in Toronto and Peel Region, 2022/2023 eTable 1. Estimates of the Incidence of iGAS Using a Denominator of Daily Homelessness (Point-in-Time Counts), and a Denominator of Annual Homelessness eReferences. eTable 2. Frequency of emm Types Among Isolates Causing iGAS in Adults, by Housing Group, TIBDN, 2022-2023, Ordered by Frequency in Persons Experiencing Homelessness, Then Housed Individuals eFigure 1. Maximum Likelihood Core Single Nucleotide Variant Phylogenetic Tree of S. pyogenes emm82 Isolates of MLST334, Comprising 49 of 53 Identified emm82 Isolates in TIBDN iGAS Surveillance Canada, January 2022–December 2023 eFigure 2. Maximum Likelihood Core Single Nucleotide Variant Phylogenetic Tree of S. pyogenes emm92 Isolates of MLST82, Comprising 39 of 40 Identified emm92 Isolates in TIBDN iGAS Surveillance Canada, January 2022–December 2023 eFigure 3. Maximum Likelihood Core Single Nucleotide Variant Phylogenetic Tree of S. pyogenes emm80 Isolates of MLST538, Comprising all 26 Identified emm80 Isolates in TIBDN iGAS Surveillance Canada, January 2022–December 2023 eFigure 4. Maximum Likelihood Core Single Nucleotide Variant Phylogenetic Tree of S. pyogenes emm74 Isolates of MLST120, Comprising all 20 Identified emm74 Isolates in TIBDN iGAS Surveillance Canada, January 2022–December 2023 [file jamanetwopen-e2557932-s001.pdf]

Supplementary Online Content

Kassee C, Dabaja Younis H, Richard L, et al; Toronto Invasive Bacterial Diseases Network. Invasive group A streptococcal disease in persons experiencing postpandemic homelessness in Canada. *JAMA Netw Open.* 2026;9(2):e2557932. doi:10.1001/jamanetworkopen.2025.57932

eMethods. Estimating the Population Experiencing Homelessness in Toronto and Peel Region, 2022/2023
eTable 1. Estimates of the Incidence of iGAS Using a Denominator of Daily Homelessness (Point-in-Time Counts), and a Denominator of Annual Homelessness
eReferences.
eTable 2. Frequency of *emm* Types Among Isolates Causing iGAS in Adults, by Housing Group, TIBDN, 2022-2023, Ordered by Frequency in Persons Experiencing Homelessness, Then Housed Individuals
eFigure 1. Maximum Likelihood Core Single Nucleotide Variant Phylogenetic Tree of *S. pyogenes emm82* Isolates of MLST334, Comprising 49 of 53 Identified *emm82* Isolates in TIBDN iGAS Surveillance Canada, January 2022–December 2023
eFigure 2. Maximum Likelihood Core Single Nucleotide Variant Phylogenetic Tree of *S. pyogenes emm92* Isolates of MLST82, Comprising 39 of 40 Identified *emm92* Isolates in TIBDN iGAS Surveillance Canada, January 2022–December 2023
eFigure 3. Maximum Likelihood Core Single Nucleotide Variant Phylogenetic Tree of *S. pyogenes emm80* Isolates of MLST538, Comprising all 26 Identified *emm80* Isolates in TIBDN iGAS Surveillance Canada, January 2022–December 2023
eFigure 4. Maximum Likelihood Core Single Nucleotide Variant Phylogenetic Tree of *S. pyogenes emm74* Isolates of MLST120, Comprising all 20 Identified *emm74* Isolates in TIBDN iGAS Surveillance Canada, January 2022–December 2023

This supplementary material has been provided by the authors to give readers additional information about their work.

© 2026 Kassee C et al. *JAMA Network Open.*

eMethods. Estimating the Population Experiencing Homelessness in Toronto and Peel Region, 2022/2023

Our base method for estimating the number of homeless persons interpolated the data from 2021 and 2024 Point-in-Time (PiT) counts in Toronto and Peel region. This method recognizes that the incubation period for iGAS is short (14 hours-10 days),[1] and assumes that the relevant risk factor is experiencing homelessness, and that risk does not vary with duration of homelessness.

The City of Toronto and Peel Region both conducted PiT counts in 2021 and 2024 (PiT counts in both jurisdictions are normally biennial, but were delayed during the pandemic), using standardized methods to perform point-in-time counts of individuals staying in municipally and provincially-administered shelters and hotels, respite sites, health and correctional facilities, as well as those staying outdoors or in encampments[2-6]. Toronto surveys reported 7,347 PEH in April 2021 and 15,418 in October 2024, while Peel's PiT counts reported 866 persons in October 2021 and 2,799 in November 2024. For each region, these figures were used as endpoints and monthly counts estimated for the intervening period, assuming a constant percentage monthly increase in the population, with mid-year populations then estimated for 2022 and 2023: 9,574 and 11,834 for Toronto, and 1,152 and 1,685 for Peel Region, for a total population of PEH of 10,726 for 2022 and 13,519 for 2023.

It is also possible to estimate the number of PEH over a 1 year period in our population. Using this estimate underestimates the true incidence, because persons not experiencing homelessness at the time of their iGAS infection, but homeless are other times during the year, will not be counted in the numerator of homeless cases. However, it provides a lower bound to the true incidence, and gives an estimate of the number of people who might need to be vaccinated each year to provide protection in the homeless population.

Toronto Shelter and Support Services (TSSS) and Peel Region report the total number of individuals who access shelter and related overnight support services annually [7-10]. Because these estimates do not include unsheltered individuals, these estimates need to be adjusted upwards by the percentage of persons identified as unsheltered. An estimate of the number of unsheltered persons can be obtained from PiT counts, a unique object multiplier approach which has been shown to result in population estimates similar to or slightly lower than other methods to detect hard-to-reach populations [11]. TSSS reported 20,700 individuals accessing shelter services in 2022, increasing to 22,000 in 2023[9,10]. Similarly, reports to Peel Regional Council cited 4,000 individuals using Peel's shelter system in 2022, and 6,007 in 2023[7,8]. PiT counts in both Toronto and Peel estimated the proportion of unsheltered persons as 10% in 2021 and 11% in 2024 [3-6]. Thus the estimated population of PEH at any time in 2022 was 22,874 in Toronto and 4,420 in Peel, while that in 2023 was 24,310 in Toronto and 6,638 in Peel.

Estimates of the incidence of iGAS using a denominator of daily homelessness (Point-in-Time counts), and a denominator of annual homelessness are shown in eTable 1 below.

© 2026 Kassee C et al. *JAMA Network Open.*

eTable 1. Estimates of the Incidence of iGAS Using a Denominator of Daily Homelessness (Point-in-Time Counts), and a Denominator of Annual Homelessness

Year	Point-in-Time population estimate	Estimate of population homeless at any time during the year	Number of iGAS cases in persons experiencing homelessness	Estimate #1: Incidence of of iGAS per 100,000 (95% CI)	Estimate #2: Incidence of iGAS per 100,000 (95% CI)
**2022**	10,726	27,294	29	270 (181-388)	106 (71-153)
**2023**	13,519	30,948	61	451 (345-580)	197 (151-253)

© 2026 Kassee C et al. *JAMA Network Open.*

eReferences.

1. Heymann DL, editor. Control of communicable diseases manual. 21 st ed. Washington, DC: American Public Health Association; 2022.
2. Housing, Infrastructure and Community, Canada. Point-in-Time Counts of Homelessness. https://housing-infrastructure.canada.ca/homelessness-sans-abri/resources-ressources/point-in-time-denombrement-ponctuel-eng.html. Accessed 4August2025
3. City of Toronto (2021) Street Needs Assessment Report. Available at https://www.toronto.ca/legdocs/mmis/2021/ec/bgrd/backgroundfile-171729.pdf. Accessed June 22, 2025.
4. City of Toronto (2024) Street Needs Assessment Report. Available at https://www.toronto.ca/wp-content/uploads/2025/07/9790-street-needs-assessment-report-2024.pdf. Accessed June 22, 2025.
5. Region of Peel (2021) Everyone Counts Peel - 2021 Community Report. Available at https://pub-peelregion.escribemeetings.com/filestream.ashx?DocumentId=24624 Accessed July 22, 2025.
6. Region of Peel (2024) Everyone Counts Peel - 2024 Community Report. Available at https://pub-peelregion.escribemeetings.com/filestream.ashx?DocumentId=38914. Accessed July 22, 2025.
7. Baird S (2023) Homelessness Policy and Programs. Available at https://pub-peelregion.escribemeetings.com/filestream.ashx?DocumentId=30190. Accessed July 29. 2025.
8. Jacques S (2025) A Coordinated Encampment Response for Peel Region, the City of Brampton, the City of Mississauga, and the Town of Caledon. Available at https://pub-peelregion.escribemeetings.com/filestream.ashx?DocumentId=39984. Accessed July 24, 2025.
9. Shelter Support and Housing Administration (SSHA) (2022) Annual Report. Available at https://www.toronto.ca/wp-content/uploads/2023/05/8dcc-2022-SSHA-Annual-ReportAODA.pdf Accessed July 25, 2025.
10. Toronto Shelter and Support Services (TSSS) (2023) Annual Report. Available at https://www.toronto.ca/wp-content/uploads/2024/05/97fb-TSSSAnnualReport2023AODA.pdf. Accessed July 24, 2025.
11. Wesson, P., Reingold, A. & McFarland, W. Theoretical and Empirical Comparisons of Methods to Estimate the Size of Hard-to-Reach Populations: A Systematic Review. *AIDS Behav* **21**, 2188–2206 (2017). DOI:10.1007/s10461-017-1678-9

© 2026 Kassee C et al. *JAMA Network Open.*

eTable 2. Frequency of *emm* Types Among Isolates Causing iGAS in Adults, by Housing Group, TIBDN, 2022-2023, Ordered by Frequency in Persons Experiencing Homelessness, Then Housed Individuals

***emm* type**	***emm* cluster**[1]	**Persons experiencing homelessness (n=90)**	**Housed Persons (n=406)**	**Total (n=496)**
49	E3	21 (23.3%)	49 (12.1%)	70 (14.1%)
82	E3	17 (18.9%)	36 (8.9%)	53 (10.7%)
92	E2	12 (13.3%)	28 (6.9%)	40 (8.1%)
74	Clade Y	11 (12.2%)	9 (2.2%)	20 (4.0%)
80	D4	9 (10.0%)	17 (4.2%)	26 (5.2%)
53	D4	5 (5.6%)	13 (3.2%)	18 (3.6%)
81	E6	3 (3.3%)	8 (2.0%)	11 (2.2%)
12	A-C4	2 (2.2%)	72 (17.7%)	74 (14.9%)
83	D4	2 (2.2%)	1 (0.2%)	3 (0.6%)
265	N/A	2 (2.2%)	1 (0.2%)	3 (0.6%)
22	E4	1 (1.1%)	8 (2.0%)	9 (1.8%)
59	E6	1 (1.1%)	2 (0.5%)	3 (0.6%)
76	E2	1 (1.1%)	4 (1.0%)	5 (1.0%)
91	D4	1 (1.1%)	1 (0.2%)	2 (0.4%)
169	E4	1 (1.1%)	4 (1.0%)	5 (1.0%)
183	E3	1 (1.1%)	0	1 (0.2%)
		emm types causing iGAS in housed persons only		
1	A-C3	0	65 (16.0%)	65 (13.1%)
89	E4	0	15 (3.7%)	15 (3.0%)
28	E4	0	13 (3.2%)	13 (2.6%)
77	E4	0	12 (2.9%)	12 (2.4%)
2	E4	0	7 (1.7%)	7 (1.4%)
11	E6	0	5 (1.2%)	5 (1.0%)
25	E3	0	3 (0.7%)	3 (0.6%)
63	E6	0	3 (0.7%)	3 (0.6%)
87	E3	0	3 (0.7%)	3 (0.6%)
5	Clade Y	0	2 (0.5%)	2 (0.4%)
9	E3	0	2 (0.5%)	2 (0.4%)
66	E2	0	2 (0.5%)	2 (0.4%)
68	E2	0	2 (0.5%)	2 (0.4%)
75	E6	0	2 (0.5%)	2 (0.4%)
90	E2	0	2 (0.5%)	2 (0.4%)
Other[2]	Multiple	0	15 (3.7%)	15 (3.0%)

[1]*emm* clusters assigned as described in Sanderson-Smith, *et al.* A systematic and functional classification of Streptococcus pyogenes that serves as a new tool for molecular typing and vaccine development. J Infect Dis. 2014;210:1325-38. doi: 10.1093/infdis/jiu260.
[2]Other isolates comprise a single isolate each of *emm* types: 3, 6, 18, 33, 48, 58, 64, 65, 73, 93, 102, 104,109, 122, 168

© 2026 Kassee C et al. *JAMA Network Open.*

eFigure 1. Maximum Likelihood Core Single Nucleotide Variant Phylogenetic Tree of *S. pyogenes emm82* Isolates of MLST334, Comprising 49 of 53 Identified *emm82* Isolates in TIBDN iGAS Surveillance Canada, January 2022–December 2023. A total of 142 sites were used in the phylogeny, including 97.8% of the core genome. Internal isolate SC23-0435-A, the first identified isolate, was used as a mapping reference and tree root. Tip node shades represent isolates from PEH (grey), housed adults not known to be PWID (white), and housed adults who are PWID (black).

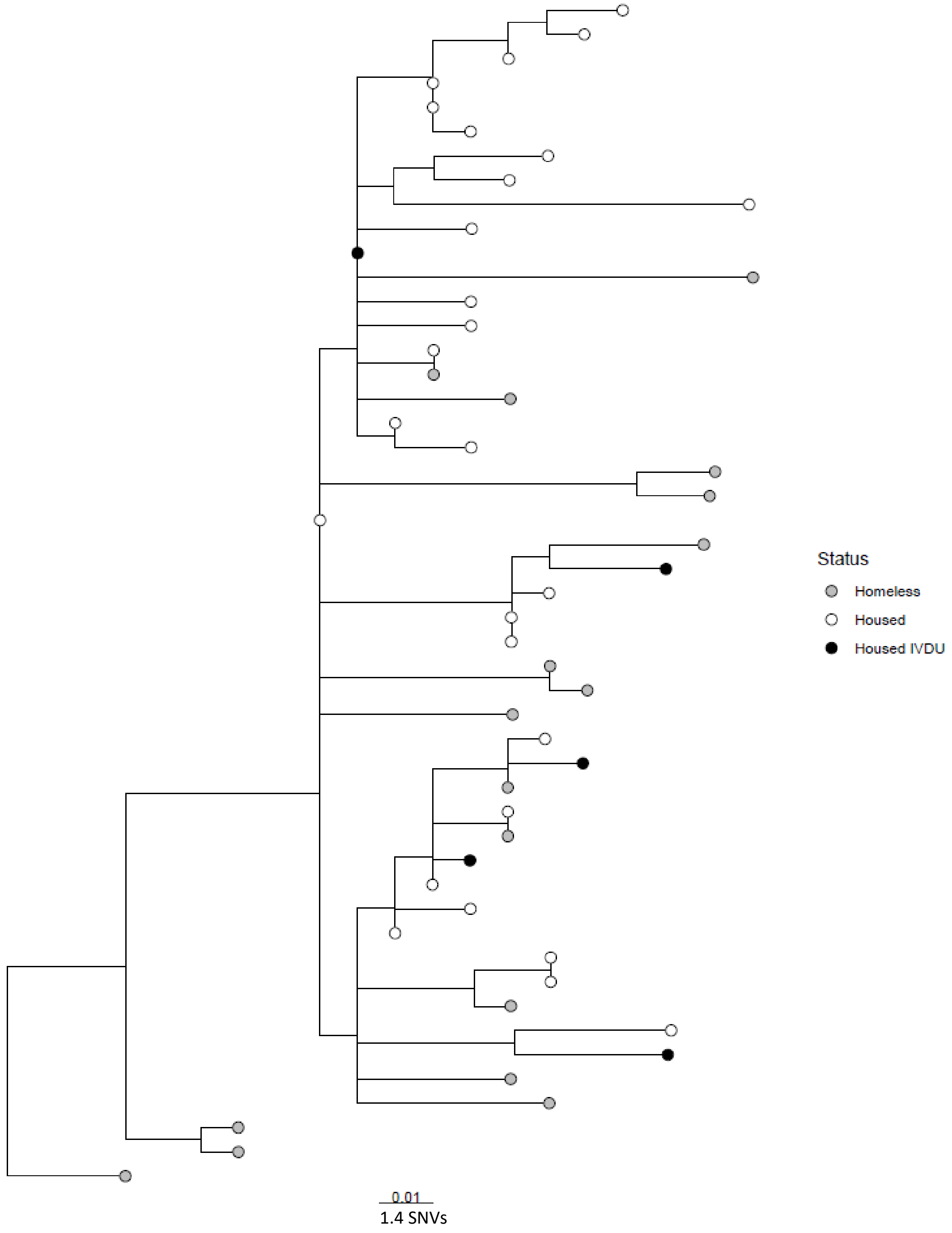

© 2026 Kassee C et al. *JAMA Network Open.*

eFigure 2. Maximum Likelihood Core Single Nucleotide Variant Phylogenetic Tree of *S. pyogenes emm92* Isolates of MLST82, Comprising 39 of 40 Identified *emm92* Isolates in TIBDN iGAS Surveillance Canada, January 2022–December 2023. A total of 93 sites were used in the phylogeny, including 96.2% of the core genome. Internal isolate SC22-1037-A, the first identified isolate, was used as a mapping reference and tree root. Tip node shades represent isolates from PEH (grey), housed adults not known to be PWID (white), and housed adults who are PWID (black).

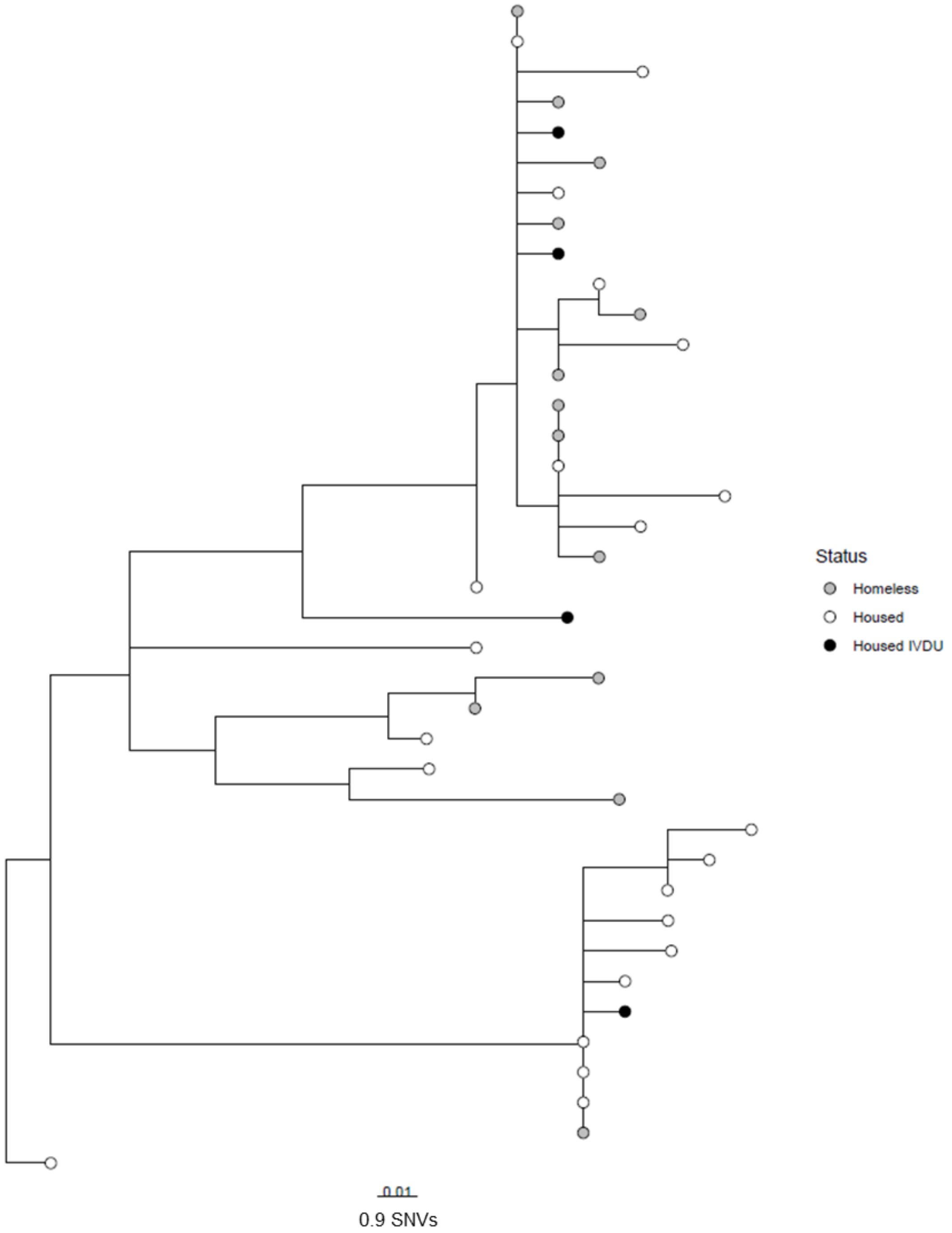

© 2026 Kassee C et al. *JAMA Network Open.*

eFigure 3. Maximum Likelihood Core Single Nucleotide Variant Phylogenetic Tree of *S. pyogenes emm80* Isolates of MLST538, Comprising all 26 Identified *emm80* Isolates in TIBDN iGAS Surveillance Canada, January 2022–December 2023. A total of 42 sites were used in the phylogeny, including 95.9% of the core genome. Internal isolate SC22-0683-A, the first identified isolate, was used as a mapping reference and tree root. Tip node shades represent isolates from PEH (grey), housed adults not known to be PWID (white), and housed adults who are PWID (black).

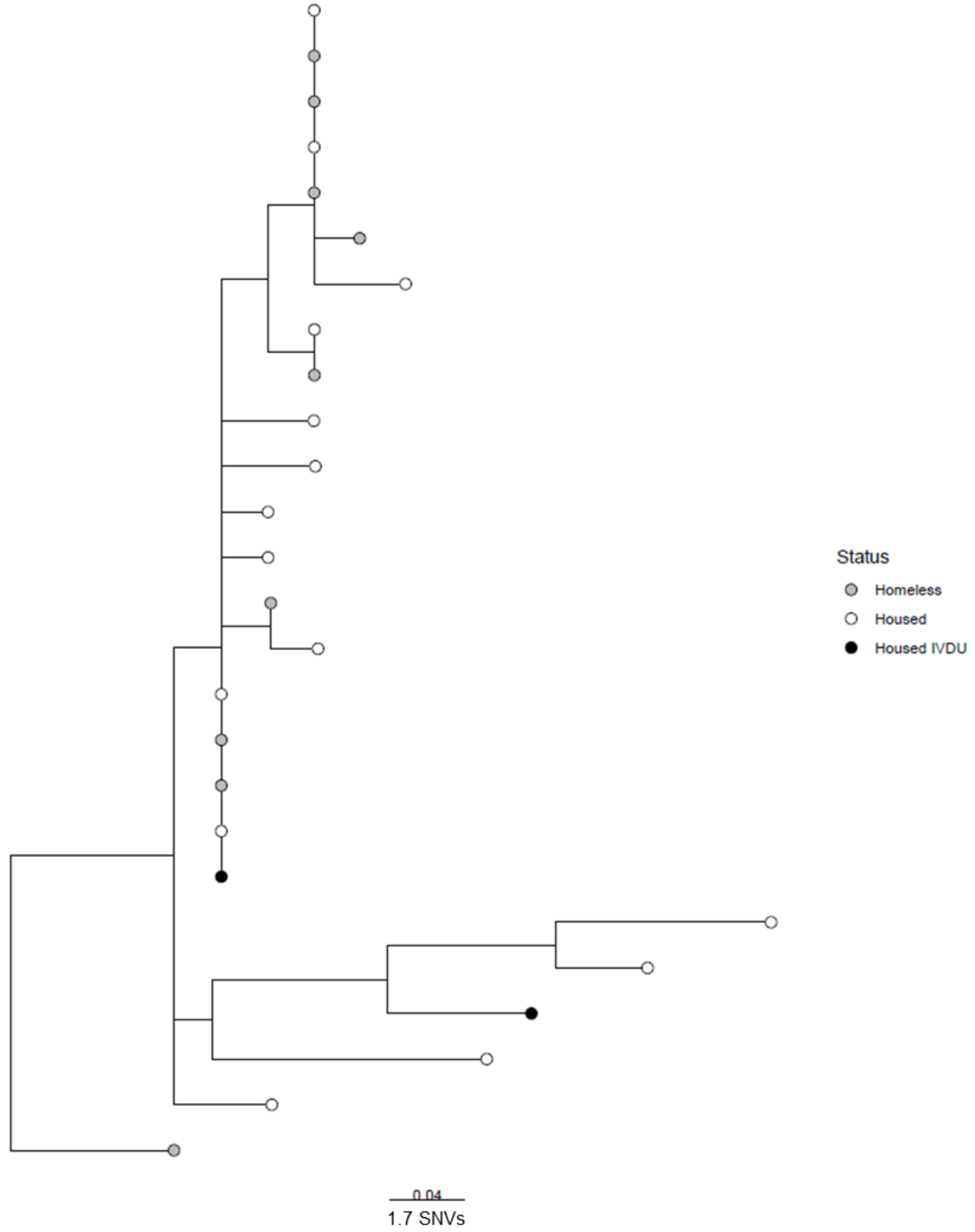

© 2026 Kassee C et al. *JAMA Network Open.*

eFigure 4. Maximum Likelihood Core Single Nucleotide Variant Phylogenetic Tree of *S. pyogenes emm74* Isolates of MLST120, Comprising all 20 Identified *emm74* Isolates in TIBDN iGAS Surveillance Canada, January 2022–December 2023. A total of 31 sites were used in the phylogeny, including 99.1% of the core genome. Internal isolate SC23-9312-A, the first identified isolate, was used as a mapping reference and tree root. Tip node shades represent isolates from PEH (grey), housed adults not known to be PWID (white), and housed adults who are PWID (black).

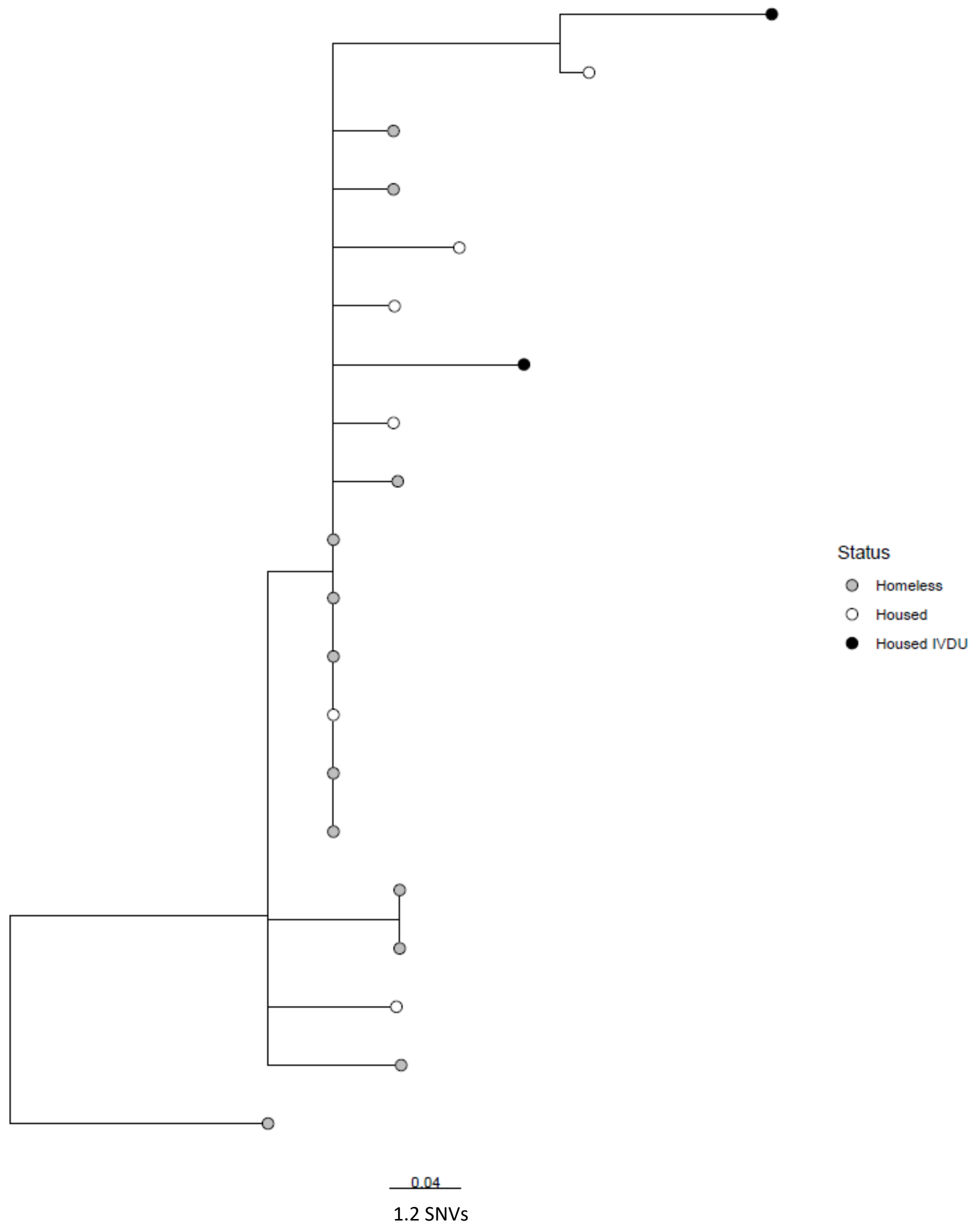

© 2026 Kassee C et al. *JAMA Network Open.*
