# Supplementary material for: Invasive Group A Streptococcal Disease in Persons Experiencing Postpandemic Homelessness in Canada
Source: JAMA Netw Open. 2026 Feb 10;9(2):e2557932. doi: 10.1001/jamanetworkopen.2025.57932 (PMC12892144; doi:10.1001/jamanetworkopen.2025.57932)
Supplement: Supplement 2. — Nonauthor Collaborators. Toronto Invasive Bacterial Diseases Network Nonauthor Collaborators [file jamanetwopen-e2557932-s002.pdf]

Supplemental Online Content: Nonauthor Collaborators
*First name, last name, and suffix (if applicable) are required and will appear in PubMed.

*Group Name(s): Toronto Invasive Bacterial Diseases Network							
***First Name and Middle Initial(s)**	***Last Name**	***Suffix (eg, Jr, III)**	Academic Degrees	Institution	Location (city, state/province, country)	Role or Contribution, eg, chair, principal investigator	Group (if more than 1 Group listed in the byline) and/or Subgroup (eg, Steering Committee)
Chingiz	Amirov		MPH	Baycrest Health Sciences	Toronto, Ontario, Canada	Site investigator	
Lee	Goneau		PhD	Dynacare	Brampton, Ontario, Canada	Site investigator	
Neil	Rau		MD	Halton Healthcare	Oakville, Ontario, Canada	Site investigator	
Deborah	Yamamura		MD	Hamilton Regional Laboratory Medici	Hamilton, Ontario, Canada	Site investigator	
Theodora	Ruijs		MD	Headwaters Healthcare Centre	Orangeville, Ontario, Canada	Site investigator	
Jennifer	Yoon		RN	Humber River Health	Toronto, Ontario, Canada	Site investigator	
Danny	Chen		MD	Mackenzie Health	Richmond Hill, Ontario Canada	Site investigator	
Jeff	Powis		MD	Michael Garron Hospital	Toronto, Ontario, Canada	Site investigator	
Amna	Faheem		MBBS	North York General Hospital	Toronto, Ontario, Canada	Co-investigator	
Kevin	Katz		MD	North York General Hospital	Toronto, Ontario, Canada	Site investigator	
Valerie	Sales		MD	Oak Valley Health	Markham, Ontario, Canada	Site investigator	
Samir	Patel		PhD	Public Health Ontario Laboratory	Toronto, Ontario, Canada	Site investigator	
Abdelbaset	Belhaj		MBBS	Scarborough Health Network	Toronto, Ontario, Canada	Site investigator	
Reena	Lovinsky		MD	Scarborough Health Network	Toronto, Ontario, Canada	Site investigator	
David	Rose		MD	Scarborough Health Network	Toronto, Ontario, Canada	Site investigator	
Christie	Vermeiren		PhD	Shared Hospital Laboratory	Toronto, Ontario, Canada	Site investigator	
Susan	Poutanen		MD	Sinai Health	Toronto, Ontario, Canada	Co-investigator	
Tony	Mazzulli		MD	Sinai Health	Toronto, Ontario, Canada	Site investigator	
Jennie	Johnstone		MD	Sinai Health	Toronto, Ontario, Canada	Co-Investigator	
Karen	Green		MSc	Sinai Health	Toronto, Ontario, Canada	Co-Investigator	
Brenda	Coleman		PhD	Sinai Health	Toronto, Ontario, Canada	Co-Investigator	
Mary Ann	MacDonald		MLT	Soldier's Memorial Hospital	Orillia, Ontario Canada	Site investigator	
Michael	Lingley		MD	Southlake Regional Health Centre	Newmarket, Ontario, Canada	Site investigator	
Jerome	Leis		MD	Sunnybrook Health Sciences Centre	Toronto, Ontario, Canada	Co-Investigator	
Andrew	Simor		MD	Sunnybrook Health Sciences Centre	Toronto, Ontario, Canada	Site investigator	
Samira	Mubareka		MD	Sunnybrook Health Sciences Centre	Toronto, Ontario, Canada	Site investigator	
Ari	Bitnun		MD	The Hospital for Sick Children	Toronto, Ontario, Canada	Co-Investigator	
Pierre-Philippe	Piche-Renaud		MD	The Hospital for Sick Children	Toronto, Ontario, Canada	Co-Investigator	
Ramzi	Fattouh		PhD	Unity Health Toronto	Toronto, Ontario, Canada	Co-investigator	
Sigmund	Krajden		MD	Unity Health Toronto	Toronto, Ontario, Canada	Site investigator	

Supplemental Online Content: Nonauthor Collaborators
*First name, last name, and suffix (if applicable) are required and will appear in PubMed.

***First Name and Middle Initial(s)**	***Last Name**	***Suffix (eg, Jr, III)**	Academic Degrees	Institution	Location (city, state/province, country)	Role or Contribution, eg, chair, principal investigator	Group (if more than 1 Group listed in the byline) and/or Subgroup (eg, Steering Committee)
Larissa	Matukas		MD	Unity Health Toronto	Toronto, Ontario, Canada	Site investigator	
Thomas	Dashwood		MD	Unity Health Toronto	Toronto, Ontario, Canada	Site investigator	
Sharon	Walmsley		MD	University Health Network	Toronto, Ontario, Canada	Site investigator	
Alainna	Jamal		MD	University of Toronto	Toronto, Ontario, Canada	Co-investigator	
Sandi	Noble		RN	Westpark Healthcare Centre	Toronto, Ontario, Canada	Site investigator	
Mona	Loutfy		MD	Women's College Hospital	Toronto, Ontario, Canada	Site investigator	
