# Supplementary material for: Invasive Group A Streptococcal Disease in Persons Experiencing Postpandemic Homelessness in Canada
Source: JAMA Netw Open. 2026 Feb 10;9(2):e2557932. doi: 10.1001/jamanetworkopen.2025.57932 (PMC12892144; doi:10.1001/jamanetworkopen.2025.57932)
Supplement: Supplement 3. — Data Sharing Statement [file jamanetwopen-e2557932-s003.pdf]

Data Sharing Statement

Kassee. Invasive Group A Streptococcal Disease in Persons Experiencing Postpandemic Homelessness in Canada. *JAMA Netw Open*. Published February 10, 2026.
doi:10.1001/jamanetworkopen.2025.57932

Data
Data available: Yes
Data types: Deidentified participant data, Data dictionary
How to access data: Requests should be sent to allison.mcgeer@sinaihealth.ca
When available: With publication

Supporting Documents
Document types: None

Additional Information
Who can access the data: Data are available on request from groups with proposals approved by a qualified Research ethics board. The proposal must be approved by TIBDN investigators and by the REB of Sinai Health System, and a data transfer agreement completed.
Types of analyses: For any purpose, with REB and TIBDN investigator approval
Mechanisms of data availability: After approval by the Research Ethics Board of the investigators and of Sinai Health, as well as by TIBDN investigators, and with a signed data transfer agreement completed.
